# Supplementary material for: Enhancement of Radiosurgical Treatment Outcome Prediction Using MRI Radiomics in Patients with Non-Small Cell Lung Cancer Brain Metastases
Source: Cancers (Basel). 2021 Aug 10;13(16):4030. doi: 10.3390/cancers13164030 (PMC8392266; doi:10.3390/cancers13164030)
Supplement: Supplementary file 1 [file cancers-13-04030-s001.zip › cancers-1236038-supplementary.pdf]

Supplementary Materials:

Enhancement of Radiosurgical Treatment Outcome Prediction Using MRI Radiomics in Patients with Non-Small Cell Lung Cancer Brain Metastases

Table S1. The formulae for the calculation of primary radiomic features.

**Intensity-based features (first order statistics)** X denotes the intensity vector with N voxels of the tumor ROIs; $\bar{X}$, the mean of **X**; **P**, the first-order histogram with N_l discrete intensity levels.			
**Feature**	**Formula**	**Feature**	**Formula**
1. Energy	$\sum_{i=1}^{N}\mathbf{X}(i)^2$	2. Entropy	$\sum_{i=1}^{N_l}\mathbf{P}(i)log_2\mathbf{P}(i)$
3. Kurtosis	$\frac{\frac{1}{N}\sum_{i=1}^{N}(\mathbf{X}(i)-\bar{X})^4}{\left(\sqrt{\frac{1}{N}\sum_{i=1}^{N}(\mathbf{X}(i)-\bar{X})^2}\right)^2}-3$	4. Maximum	$\max(\mathbf{X})$
5. Mean	$\frac{1}{N}\sum_{i=1}^{N}\mathbf{X}(i)$	6. Mean absolute deviation	$\frac{1}{N}\sum_{i=1}^{N}\text{abs}(\mathbf{X}(i)-\bar{X})$
7. Median	$\text{median}(\mathbf{X})$	8. First quartile	Value that splits off the lowest 25% of data from the highest 75%
9. Third quartile	Value that splits off the highest 25% of data from the lowest 75%	10. Minimum	$\min(\mathbf{X})$
11. Range	$\max(\mathbf{X})-\min(\mathbf{X})$	12. Root mean square (RMS)	$\sqrt{\frac{\sum_{i=1}^{N}\mathbf{X}(i)^2}{N}}$
13. Skewness	$\frac{\frac{1}{N}\sum_{i=1}^{N}(\mathbf{X}(i)-\bar{X})^3}{\left(\sqrt{\frac{1}{N}\sum_{i=1}^{N}(\mathbf{X}(i)-\bar{X})^2}\right)^3}$	14. Standard deviation	$\sqrt{\frac{1}{N}\sum_{i=1}^{N}(\mathbf{X}(i)-\bar{X})^2}$
15. Uniformity	$\sum_{i=1}^{N_l}\mathbf{P}(i)^2$	16. Variance	$\frac{1}{N}\sum_{i=1}^{N}(\mathbf{X}(i)-\bar{X})^2$
**Shape- and Size-based features** V, tumor volume; A, surface area of the volume			
17. Compactness 1	$\frac{V}{\sqrt{\pi}A^{3/2}}$	18. Compactness 2	$36\pi\frac{V^2}{A^3}$
19. Maximum 3D diameter	The largest pairwise Euclidean distance between voxels on the surface of the tumor volume.	20. Spherical disproportion	$\frac{A}{4\pi R^2}$
21. Sphericity	$\frac{\pi^{\frac{1}{3}}(6V)^{\frac{2}{3}}}{A}$	22. Surface area	$A=\sum_{i=1}^{Ns}\frac{1}{2}\lvert a_ib_i\times a_ic_i\rvert$ Ns, total number of triangles covering the surface; a, b, and c, triangle vertices
23. Surface to volume ratio	$\frac{A}{V}$	24. Volume	Number of pixels in the tumor region multiplied by the voxel size
**Textural features (gray-level co-occurrence matrix based features)** $\mathbf{P}(\delta,\alpha)$, co-occurrence matrix for an arbitrary distance δ and direction α; N_g, number of discrete intensity levels in the image; p_x(i), marginal row probabilities; p_y(i), marginal column probabilities; μ_x, mean of p_x; μ_y, mean of p_y; σ_x, standard deviation of p_x; σ_y, standard deviation of p_y; HXY, entropy of **P**; HX, entropy of p_x; HY, entropy of p_y; $p_{x+y}(k)=\sum_{i=1}^{N_g}\sum_{j=1}^{N_g}\mathbf{P}(i,j),\, i+j=k, k=2,3,\ldots,2N_g;$ $p_{x-y}(k)=\sum_{i=1}^{N_g}\sum_{j=1}^{N_g}\mathbf{P}(i,j),\, \lvert i-j\rvert=k, k=0,1,\ldots,N_g-1;$ $\text{HX}=-\sum_{i=1}^{N_g}p_x(i)\log_2(p_x(i)),\ \text{HY}=-\sum_{i=1}^{N_g}p_y(i)\log_2(p_y(i));$ $\text{HXY1}=-\sum_{i=1}^{N_g}\sum_{j=1}^{N_g}\mathbf{P}(i,j)\log_2(p_x(i)p_y(j)),\ \text{HXY2}=-\sum_{i=1}^{N_g}\sum_{j=1}^{N_g}p_x(i)p_y(j)\log_2(p_x(i)p_y(j))$			
25. Autocorrelation	$\sum_{i=1}^{N_g}\sum_{j=1}^{N_g}ij\mathbf{P}(i,j)$	26. Cluster Prominence	$\sum_{i=1}^{N_g}\sum_{j=1}^{N_g}[\boldsymbol{i}+\boldsymbol{j}-\boldsymbol{\mu_x}-\boldsymbol{\mu_y}]^4\mathbf{P}(i,j)$
27. Cluster Shade	$\sum_{i=1}^{N_g}\sum_{j=1}^{N_g}[\boldsymbol{i}+\boldsymbol{j}-\boldsymbol{\mu_x}-\boldsymbol{\mu_y}]^3\mathbf{P}(i,j)$	28. Cluster Tendency	$\sum_{i=1}^{N_g}\sum_{j=1}^{N_g}[\boldsymbol{i}+\boldsymbol{j}-\boldsymbol{\mu_x}-\boldsymbol{\mu_y}]^2\mathbf{P}(i,j)$
29. Contrast	$\sum_{i=1}^{N_g}\sum_{j=1}^{N_g}\lvert\boldsymbol{i}-\boldsymbol{j}\rvert^2\mathbf{P}(i,j)$	30. Correlation	$\sum_{i=1}^{N_g}\sum_{j=1}^{N_g}\frac{ij\mathbf{P}(i,j)-\mu_x(i)\mu_y(j)}{\sigma_x(i)\sigma_y(j)}$

31. Difference entropy	$\sum_{i=0}^{N_g-1} p_{x-y}(i) log_2[p_{x-y}(i)]$	32. Dissimilarity	$\sum_{i=1}^{N_g}\sum_{j=1}^{N_g}\|\boldsymbol{i-j}\|\mathbf{P}(i,j)$
33. Energy	$\sum_{i=1}^{N_g}\sum_{j=1}^{N_g}[\mathbf{P}(i,j)]^{\mathbf{2}}$	34. Entropy (*HXY*)	$-\sum_{i=1}^{N_g}\sum_{j=1}^{N_g}\mathbf{P}(i,j)\log_2(\mathbf{P}(i,j))$
35. Homogeneity 1	$\sum_{i=1}^{N_g}\sum_{j=1}^{N_g}\frac{\mathbf{P}(i,j)}{1+\|i-j\|}$	36. Homogeneity 2	$\sum_{i=1}^{N_g}\sum_{j=1}^{N_g}\frac{\mathbf{P}(i,j)}{1+\|i-j\|^2}$
37. Informational measure of correlation 1	$\frac{HXY-HXY1}{\max(HX,HY)}$	38. Informational measure of correlation 2	$\sqrt{1-e^{-2(HXY2-HXY)}}$
39. Inverse Difference Moment Normalized	$\sum_{i=1}^{N_g}\sum_{j=1}^{N_g}\frac{\mathbf{P}(i,j)}{1+\left(\frac{\|i-j\|^2}{N^2}\right)}$	40. Inverse Difference Normalized	$\sum_{i=1}^{N_g}\sum_{j=1}^{N_g}\frac{\mathbf{P}(i,j)}{1+\left(\frac{\|i-j\|}{N}\right)}$
41. Inverse variance	$\sum_{i=1}^{N_g}\sum_{j=1}^{N_g}\frac{\mathbf{P}(i,j)}{\|i-j\|^2}, i \neq j$	42. Maximum Probability	$\max(\mathbf{P}(i,j))$
43. Sum average	$\sum_{i=2}^{2N_g}[i\mathbf{P}_{x+y}(i)]$	44. Sum entropy	$-\sum_{i=2}^{2N_g}\mathbf{P}_{x+y}(i) log_2[\mathbf{P}_{x+y}(i)]$
45. Variance	$\sum_{i=1}^{N_g}\sum_{j=1}^{N_g}(i-\mu)^2\mathbf{P}(\mathbf{i},\mathbf{j})$		
**Textural features (gray-level run-length matrix based features)** $p(i,j\|\theta)$, (*i,j*)th entry in the given run-length matrix *p* for a direction *θ*; N_g, number of discrete intensity levels in the image; N_r, number of different run lengths			
46. Short Run Emphasis	$\frac{\sum_{i=1}^{N_g}\sum_{j=1}^{N_r}\left[\frac{p(i,j\|\theta)}{j^2}\right]}{\sum_{i=1}^{N_g}\sum_{j=1}^{N_r}p(i,j\|\theta)}$	47. Long Run Emphasis	$\frac{\sum_{i=1}^{N_g}\sum_{j=1}^{N_r}j^2 p(i,j\|\theta)}{\sum_{i=1}^{N_g}\sum_{j=1}^{N_r}p(i,j\|\theta)}$
48. Gray Level Non-Uniformity	$\frac{\sum_{i=1}^{N_g}\left[\sum_{j=1}^{N_r}p(i,j\|\theta)\right]^2}{\sum_{i=1}^{N_g}\sum_{j=1}^{N_r}p(i,j\|\theta)}$	49. Run Length Non-Uniformity	$\frac{\sum_{j=1}^{N_r}\left[\sum_{i=1}^{N_g}p(i,j\|\theta)\right]^2}{\sum_{i=1}^{N_g}\sum_{j=1}^{N_r}p(i,j\|\theta)}$
50. Run Percentage	$\sum_{i=1}^{N_g}\sum_{j=1}^{N_r}\frac{p(i,j\|\theta)}{N_p}$	51. Low Gray Level Run Emphasis	$\frac{\sum_{i=1}^{N_g}\sum_{j=1}^{N_r}\left[\frac{p(i,j\|\theta)}{i^2}\right]}{\sum_{i=1}^{N_g}\sum_{j=1}^{N_r}p(i,j\|\theta)}$
52. High Gray Level Run Emphasis	$\frac{\sum_{i=1}^{N_g}\sum_{j=1}^{N_r}i^2 p(i,j\|\theta)}{\sum_{i=1}^{N_g}\sum_{j=1}^{N_r}p(i,j\|\theta)}$	53. Short Run Low Gray Level Emphasis	$\frac{\sum_{i=1}^{N_g}\sum_{j=1}^{N_r}\left[\frac{p(i,j\|\theta)}{i^2 j^2}\right]}{\sum_{i=1}^{N_g}\sum_{j=1}^{N_r}p(i,j\|\theta)}$
54. Short Run High Gray Level Emphasis	$\frac{\sum_{i=1}^{N_g}\sum_{j=1}^{N_r}\left[\frac{p(i,j\|\theta)i^2}{j^2}\right]}{\sum_{i=1}^{N_g}\sum_{j=1}^{N_r}p(i,j\|\theta)}$	55. Long Run Low Gray Level Emphasis	$\frac{\sum_{i=1}^{N_g}\sum_{j=1}^{N_r}\left[\frac{p(i,j\|\theta)j^2}{i^2}\right]}{\sum_{i=1}^{N_g}\sum_{j=1}^{N_r}p(i,j\|\theta)}$
56. Long Run High Gray Level Emphasis	$\frac{\sum_{i=1}^{N_g}\sum_{j=1}^{N_r}i^2 j^2 p(i,j\|\theta)}{\sum_{i=1}^{N_g}\sum_{j=1}^{N_r}p(i,j\|\theta)}$		
**Textural features (local binary pattern based features)** **X** denotes the vector of local binary pattern with *N* voxels in the tumor ROIs. The local binary pattern was estimated based on the relations of center pixel with 8 neighbors; $\bar{X}$, the mean of **X**; **P**, the first-order histogram with N_l discrete intensity levels. Equations #1 to 16 (first order statistics) were then applied to yield 16 local binary pattern based features.			

Table S2. Twenty-five radiomic features with the most significant differences between stable and progression local tumor control.

Image Contrast	Wavelet Filtering	Radiomics Type	Feature Name	Local tumor control	
				Good	Poor
T1w	none	Texture-GLCM	Informational measure of correlation 1 (IMC1)	-0.49±0.54	0.49±1.12
T1w	LLL	Texture-GLCM	Informational measure of correlation 1 (IMC1)	-0.47±0.55	0.48±1.13
T1w	LLH	Texture-GLCM	Homogeneity 1	-0.52±0.46	0.53±1.13
T1w	LLH	Texture-GLCM	Informational measure of correlation 1 (IMC1)	-0.51±0.53	0.52±1.10
T1w	LHL	Texture-GLCM	Informational measure of correlation 1 (IMC1)	-0.51±0.49	0.52±1.12
T1w	LHH	Texture-GLCM	Informational measure of correlation 1 (IMC1)	-0.48±0.51	0.49±1.14
T1w	HLL	Texture-GLCM	Informational measure of correlation 1 (IMC1)	-0.52±0.46	0.53±1.12
T1w	HLH	Texture-GLCM	Informational measure of correlation 1 (IMC1)	-0.49±0.48	0.50±1.14
T1w	HHL	Texture-GLCM	Informational measure of correlation 1 (IMC1)	-0.54±0.45	0.55±1.11
T1w	HHH	Texture-GLCM	Informational measure of correlation 1 (IMC1)	-0.49±0.53	0.50±1.12
T1c	HLH	Texture-GLCM	Informational measure of correlation 1 (IMC1)	-0.50±0.48	0.51±1.13
T1c	none	Texture-GLCM	Informational measure of	-0.50±0.49	0.52±1.12

			correlation 1 (IMC1)		
T1c	LLL	Texture-GLCM	Informational measure of correlation 1 (IMC1)	-0.51±0.46	0.52±1.13
T1c	HLL	Texture-GLCM	Informational measure of correlation 1 (IMC1)	-0.48±0.49	0.49±1.15
T1c	HHH	Texture-GLCM	Informational measure of correlation 1 (IMC1)	-0.51±0.47	0.52±1.13
T1c	LHL	Texture-GLCM	Informational measure of correlation 1 (IMC1)	-0.49±0.48	0.50±1.14
T1c	LHH	Texture-GLCM	Informational measure of correlation 1 (IMC1)	-0.49±0.44	0.50±1.16
T1c	LHH	Texture-GLCM	Informational measure of correlation 1 (IMC1)	-0.48±0.50	0.49±1.14
T2w	HHH	Texture-GLCM	Informational measure of correlation 1 (IMC1)	-0.47±0.60	0.48±1.10
T2w	HHH	Texture-GLCM	Informational measure of correlation 1 (IMC1)	-0.47±0.57	0.48±1.11
T2w	LLL	Texture-GLCM	Informational measure of correlation 1 (IMC1)	-0.49±0.53	0.46±1.15
T2w	LLL	Texture-GLCM	Informational measure of correlation 1 (IMC1)	-0.50±0.50	0.51±1.13
T2w	HLH	Texture-GLCM	Informational measure of correlation 1 (IMC1)	-0.49±0.52	0.50±1.13
T2w	HLH	Texture-GLCM	Informational measure of correlation 1 (IMC1)	-0.49±0.56	0.50±1.10
T2w	LLH	Texture-GLCM	Informational measure of correlation 1 (IMC1)	-0.50±0.53	0.51±1.11

GLCM: gray-level co-occurrence matrix. In the column of wavelet filtering, L represents a low-pass filter, and H represents a high-pass filter. The combination of L and H letters stands for the filter type applied to the three image axes in order.

Table S3. Twenty-five radiomic features with the most significant differences between better and poorer overall survival.

Image Contrast	Wavelet Filtering	Radiomics Type	Feature Name	Survival > 12.2 months	Survival < 12.2 months
T1w	LLL	Histogram	Standard deviation	0.30±1.13	-0.31±0.74
T1w	LLH	Histogram	Standard deviation	0.25±1.17	-0.26±0.72
T1w	HLL	Histogram	Standard deviation	0.35±1.11	-0.36±0.73
T1w	LLL	Histogram	Maximum	0.32±1.03	-0.33±0.86
T1w	LLH	Histogram	Mean absolute deviation	0.24±1.15	-0.25±0.75
T1w	HLL	Histogram	Variance	0.35±1.15	-0.36±0.65
T2w	LLL	Histogram	Minimum	-0.15±1.02	0.16±0.97
T1w	HLL	Histogram	Mean absolute deviation	0.34±1.13	-0.35±0.71
T1w	LLH	Histogram	Minimum	-0.30±1.17	0.31±0.67
T1c	LLH	Texture-GLCM	Contrast	0.17±1.23	-0.18±0.66
T1c	LLH	Texture-GLCM	Inverse Difference Moment Normalized (IDMN)	-0.17±1.20	0.17±0.72
T1c	LLH	Texture-GLCM	Variance	0.08±1.04	-0.08±0.97
T1w	LHH	Texture-GLCM	Cluster Tendency	0.19±1.17	-0.20±0.76
T1w	LHH	Texture-GLCM	Sum variance	0.19±1.17	-0.20±0.76
T1c	HHL	Histogram	Variance	0.14±0.86	-0.14±1.12
T2w	LLH	Texture-GLCM	Correlation	-0.08±1.07	0.09±0.93
T1w	HLL	Texture-GLCM	Correlation	0.01±0.96	-0.01±1.06
T1w	HLH	Histogram	Standard deviation	0.25±1.04	-0.25±0.91
T1w	LLL	Texture-LBP	First quartile	0.15±1.08	-0.15±0.90
T1w	HLH	Histogram	Maximum	0.22±1.14	-2317±0.79
T1w	HLL	Histogram	Range	0.15±1.15	-0.15±0.81
T1w	LHH	Histogram	Mean absolute deviation	0.36±0.99	-0.37±0.88
T1w	LHH	Histogram	Standard deviation	0.36±0.99	-0.37±0.88
T1w	HHL	Texture-GLRLM	Long Run High Gray-Level Emphasis (LRHGLE)	-0.02±1.22	0.02±0.73
T1w	none	Histogram	Maximum	0.38±1.08	-0.39±0.74

GLCM: gray-level co-occurrence matrix, **GLRLM**: gray-level run-length matrix. **LBP**: local binary patterns. In the column of wavelet filtering, L represents a low-pass filter, and H represents a high-pass filter. The combination of L and H letters stands for the filter type applied to the three image axes in order.

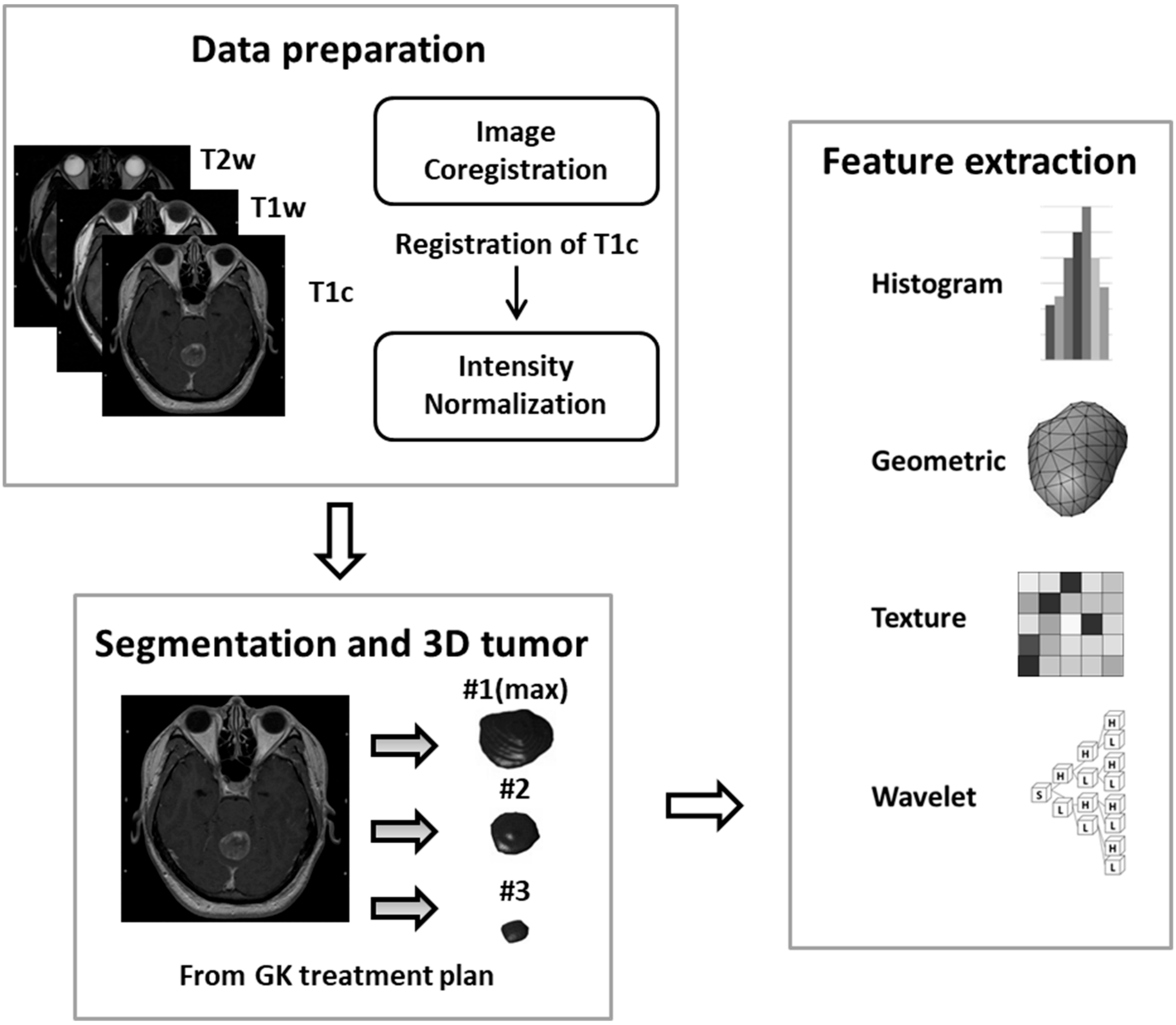

Figure S1. Processing flow of radiomics analysis**.** Axial T1w and T2w images are co-registered to the T1c images followed by the intensity normalization. 3D lesion segmentation of BMs (ROIs) are determined based on the consensus of a multidisciplinary team for the treatment planning of GKRS. Finally, radiomic features are extracted from 3D tumor regions, including histogram, geometric, textural features with the wavelet image decomposition.

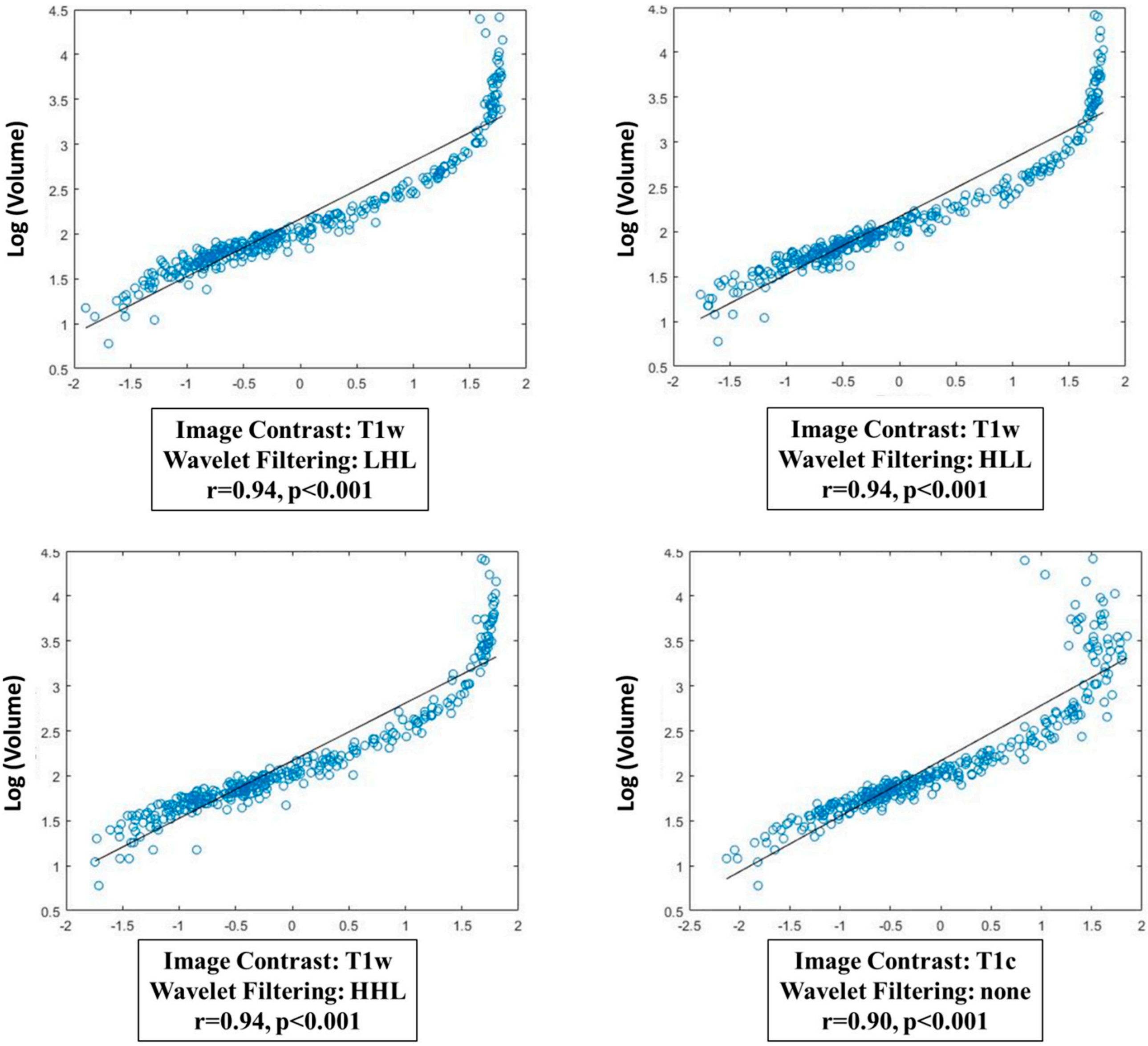

Figure S2. Correlations analysis between the four selected radiomic features (informational measure of correlation 1, IMC1) and tumor volume.
